# Calcium-sensing stromal interaction molecule 2 upregulates nuclear factor of activated T cells 1 and transforming growth factor-β signaling to promote breast cancer metastasis

**DOI:** 10.1186/s13058-019-1185-1

**Published:** 2019-08-29

**Authors:** Yutian Miao, Qiang Shen, Siheng Zhang, Hehai Huang, Xiaojing Meng, Xianchong Zheng, Zhuocheng Yao, Zhanxin He, Sitong Lu, Chunqing Cai, Fei Zou

**Affiliations:** 1 Department of Occupational Health and Occupational Medicine, School of Public Health, Southern Medical University, Guangzhou, 510515 Guangdong China; 2 Department of Clinical Cancer Prevention, The University of Texas MD Anderson Cancer Center, Houston, TX USA

**Keywords:** STIM2, Breast cancer, EMT, Metastasis, NFAT1

## Abstract

**Background:**

Stromal interaction molecule (STIM) 2 is a key calcium-sensing molecule that regulates the stabilization of calcium ions (Ca^2+^) and therefore regulates downstream Ca^2+^-associated signaling and cellular events. We hypothesized that STIM2 regulates epithelial-mesenchymal transition (EMT) to promote breast cancer metastasis.

**Methods:**

We determined the effects of gain, loss, and rescue of STIM2 on cellular motility, levels of EMT-related proteins, and secretion of transforming growth factor-β (TGF-β). We also conducted bioinformatics analyses and in vivo assessments of breast cancer growth and metastasis using xenograft models.

**Results:**

We found a significant association between STIM2 overexpression and metastatic breast cancer. STIM2 overexpression activated the nuclear factor of activated T cells 1 (NFAT1) and TGF-β signaling. Knockdown of STIM2 inhibited the motility of breast cancer cells by inhibiting EMT via specific suppression of NFAT1 and inhibited mammary tumor metastasis in mice. In contrast, STIM2 overexpression promoted metastasis. These findings were validated in human tissue arrays of 340 breast cancer samples for STIM2.

**Conclusion:**

Taken together, our results demonstrated that STIM2 specifically regulates NFAT1, which in turn regulates the expression and secretion of TGF-β1 to promote EMT in vitro and in vivo, leading to metastasis of breast cancer.

**Electronic supplementary material:**

The online version of this article (10.1186/s13058-019-1185-1) contains supplementary material, which is available to authorized users.

## Background

Metastasis accounts for around 90% of breast cancer-associated mortality [1–3]. In the initial step of metastasis, cancer cells acquire an invasive phenotype, epithelial-mesenchymal transition (EMT), which enables them to invade, migrate, and disseminate [4–9]. Many transcription factors, including Snail, Twist, and Zeb family members, are involved in mediating EMT [10]. However, despite considerable progress in understanding how EMT is modulated, the regulatory mechanisms by which EMT contributes to breast cancer metastasis are still far from clear.

STIM1 and STIM2 are members of the stromal interaction molecule (STIM) family, which are key components of store-operated Ca^2+^ channels. STIMs serve as Ca^2+^ sensors on the endoplasmic reticulum (ER) and contribute to store-operated calcium entry (SOCE) by gating and opening Orai channels in the plasma membrane when Ca^2+^ in the ER is depleted [11, 12]. Over the last decade, accumulating evidence has suggested that STIM1, in association with Orai1-mediated SOCE, is involved in cancer progression [13, 14] by regulating cellular motility to affect focal adhesion turnover [15, 16]. STIM1 was also shown to play a role in exogenous transforming growth factor (TGF)-β-induced EMT in breast cancer cells [17]. Intriguingly, STIM2 was shown to regulate SOCE with a sharper Ca^2+^-sensing capacity compared with the “slow” action of STIM1; thus, STIM2 can activate Ca^2+^ influx upon a smaller decrease of Ca^2+^ concentrations in ER. This may allow precise regulation of basal cytosolic Ca^2+^ concentration and subsequent cellular events such as cellular motility, particularly in cancer metastasis [18]. However, the precise role of STIM2 in Ca^2+^ sensing, gating rate limiting, and subsequent regulation of cellular motility, EMT, and metastasis—and how this role may differ from that of STIM1—remains unclear.

In this study, we aimed to determine STIM2’s role in regulating EMT and promoting breast cancer metastasis by identifying its specific downstream targets. Surprisingly, unlike a previous report [17], we found that STIM1 does not promote EMT in breast cancer cells. However, STIM2 promotes breast cancer metastasis via regulating the expression of the transcription factor nuclear factor of activated T cells 1 (NFAT1) and its translocation to the TGF-β1 promoter, leading to upregulated expression and secretion of TGF-β1.

## Materials and methods

### Cell lines and cultures

Human breast cancer cell lines T-47D, BT-474, MCF7, MDA-MB-231, and BT-549 were purchased from ATCC. Cells were grown in high-glucose Dulbecco’s modified Eagle’s medium with 10% fetal bovine serum and 1% penicillin/streptomycin at 37 °C in a humidified atmosphere of 5% CO_2_ and 95% air.

### Lentiviral infection and generation of stable expression cell lines

STIM1, STIM2, and NFAT1 stable knockdown (STIM1-SH, STIM2-SH, and NFAT1-SH) and overexpressing (STIM1-OE, STIM2-OE, and NFAT1-OE) clones were generated using commercially packaged lentiviral vectors (Hanbio; Genechem). Control clones (NC) were established using an empty lentiviral vector. Lentiviral infection and selection of stable expression clones were performed according to the manufacturer’s instructions. Briefly, MDA-MB-231 wild type was incubated with 5 μg/mL polybrene for 30 min at 37 °C, then infected with purified lentiviruses for 24 h. The culture medium containing lentiviruses was then replaced with fresh medium and, 48 h later, supplemented with 1 μg/mL puromycin for 24 h. Western blotting was used to confirm stable knockdown and overexpression of the genes for at least 6 passages in culture.

### Transwell migration assay

The effects of knockdown or overexpression of STIM1, STIM2, or NFAT1 on the migration of breast cancer cells were determined using Transwell assays. The bottom chambers of a 24-well Transwell plate containing Costar filters with a pore size of 8 μm (Corning) were filled with culture medium containing 10% fetal bovine serum, and 5 × 10^4^ cells suspended in serum-free medium were placed on the inserts in the upper chambers for culture at 37 °C in 5% CO_2_. After incubation for 12 h, the cells on the upper filter surface were removed using a cotton swab. Cells that had penetrated the filter and attached to the lower filter surface were fixed with 4% formaldehyde and stained with 0.5% crystal violet. Images of the stained cells were acquired under a × 20 objective microscope. Migrated cells were quantified by using Image Pro Plus 6.0 software (Media Cybernetics).

### RNA extraction and real-time quantitative PCR

Total RNA was extracted from cultured cells using RNAiso Plus (Takara Bio), and 1-μg aliquots of total RNA were subjected to cDNA synthesis using a PrimeScript RT reagent Kit (Takara Bio) according to the product manual. mRNA expression was analyzed with real-time quantitative PCR (qRT-PCR) using a SYBR Premix Ex Taq kit (Takara Bio) and a LightCycler 96 Real-Time PCR System (Roche) for 40 cycles. The qRT-PCR analysis was repeated for at least 3 independent experiments using different RNA samples. Expression levels were calculated relative to those of GAPDH using the 2^−ΔΔCt^ method. The sequences of the primers used for amplification are listed in the Additional file 1.

### Western blot analysis

Cells were lysed in radioimmunoprecipitation assay buffer (KeyGEN), and 40 μg of denatured protein extracts was resolved by 8–15% sodium dodecyl sulfate-polyacrylamide gel electrophoresis and transferred to polyvinylidene difluoride membranes. The membranes were blocked in 5% bovine serum albumin dissolved in Tris-buffered saline containing 0.1% Tween 20 (TBST) for 1 h at room temperature. The membranes were then incubated with specific primary antibodies (STIM1, 1:1000, cat # A7411, ABclonal; STIM2, 1:1000, cat # PRS4125, Sigma; NFAT1, 1:1000, cat #22023-1-AP, Proteintech; E-cadherin, 1:1000, cat # 14472S, CST; vimentin, 1:1000, cat # 5741 T, CST; histone H3, 1:5000, cat # 17168-1-AP, Proteintech; and β-actin, 1:15,000, cat # RM2001, RAY Antibody) diluted in TBST containing 3% bovine serum albumin overnight at 4 °C. Peroxidase-conjugated anti-mouse (1:15,000, cat # A0168, Sigma) and anti-rabbit (1:15,000, cat # A0545, Sigma) secondary antibodies were used. The blots were quantified by using a LI-COR Odyssey infrared imaging system.

### Tissue microarray and immunohistochemistry (IHC)

Paraffin-embedded human breast cancer tissue microarrays BR8011 and BR10010d were obtained from US Biomax (http://www.alenabio.com/welcome?lang=zh_CN) and used for IHC analyses. IHC was performed by the company which provided the tissue microarrays. Missing or inconclusive cores were removed before analysis. The intensity of IHC staining on the array was determined according to the following criteria: (−) represents non-staining, weak positive (+) represents light-brown staining, (++) represents brown staining, and (+++) represents dark brown staining. Proportion of positively stained cells in an area was determined according to the following criteria: (+) refers to the number of positive cells below 25%, (++) refers to 25–49%, and (+++) refers more than 50%. Finally, the results of qualitative and semi-quantitative coloring intensity were obtained. At least 5–10 HPFs were randomly observed, and their mean values were determined. An attending pathologist confirmed the histological diagnosis of breast cancer for the tissue arrays.

### Tumor xenografts and in vivo metastasis studies

Six-week-old female Balb/c-nu mice were obtained and maintained in the animal facility of the Laboratory Animal Center of Southern Medical University (Guangzhou, China). Procedures involving animals and their care were conducted in accordance with a protocol approved by Southern Medical University’s Experimental Animal Ethics Committee (Authorization No. 2016038). For xenograft tumor assay, cells with knockdown or overexpression of STIM2 (NC, STIM2-SH, or STIM2-OE) (5 × 10^6^) were resuspended in 100 μl of PBS and were inoculated into the mammary fat pad of mice (*n* = 7 per group). Tumor growth was evaluated by monitoring tumor volume (TV = length × width^2^ × 0.5) every 3 days for 8 weeks.

The same cell lines (2.5 × 10^5^) were injected via tail vein into female nude mice (*N* = 7) to develop experimental lung and liver metastases. After 42 days, the mice were euthanized, and their lungs and livers were evaluated for the presence of metastatic tumors. Paraffin sections (4 μm) of harvested lungs and livers were stained with hematoxylin and eosin (H&E), and images were acquired under a × 20 objective. The number of metastatic nodules was counted.

### Immunofluorescence

Cells were grown in a confocal dish (NEST). Images were acquired under a × 60 oil objective lens on an Olympus FV1000-IX71 laser scanning confocal microscope.

### Ingenuity Pathway Analysis

To assess changes in active molecular signaling pathways after knockdown of NFAT1, we analyzed canonical pathways and gene network interactions with Ingenuity Pathway Analysis (IPA). *P* values were calculated by using Ingenuity Pathways Knowledge Base-dependent Fisher exact test.

### Reporter constructs and double luciferase reporter gene assessment

The transcription factor binding site of NFAT1 on the promoter of TGF-β1 was predicted by using the JASPAR database of transcription factor binding profiles at http://jaspar.genereg.net/. Dual-luciferase reporter assays were performed using a Promega Dual-Luciferase Reporter Assay System (E1910) according to the manufacturer’s instructions. The reporter vector of human TGF-β1 promoter region was purchased from the Genechem Biotechnology Company (Shanghai, China). Technical details are included in the Additional file 1.

### Chromatin immunoprecipitation assay

Chromatin immunoprecipitation (ChIP) assays were performed using a ChIP-IT Express Kit (Active Motif) according to the manufacturer’s protocol.

### Enzyme-linked immunosorbent assay (ELISA)

The ELISA Kit (KGEHC107b) from KeyGEN BioTECH company was used to test the TGF-β1 in culture.

### Statistical analysis

Statistical analyses were performed using SPSS software version 16.0. *P* values of less than 0.05 were considered statistically significant. Means were calculated using at least 3 biological replicates unless otherwise stated. The Student *t* test was used for comparisons of 2 independent groups. Differences among groups were determined by one-way analysis of variance with repeated measures, followed by the Bonferroni post hoc test. In addition, the *χ*^2^ test was applied to analyze associations between STIM1 or STIM2 expression and clinicopathological status for the microarray data.

## Results

### Gain or loss of STIM2 affects breast cancer xenograft growth and metastasis in vivo

**Fig. 1:**
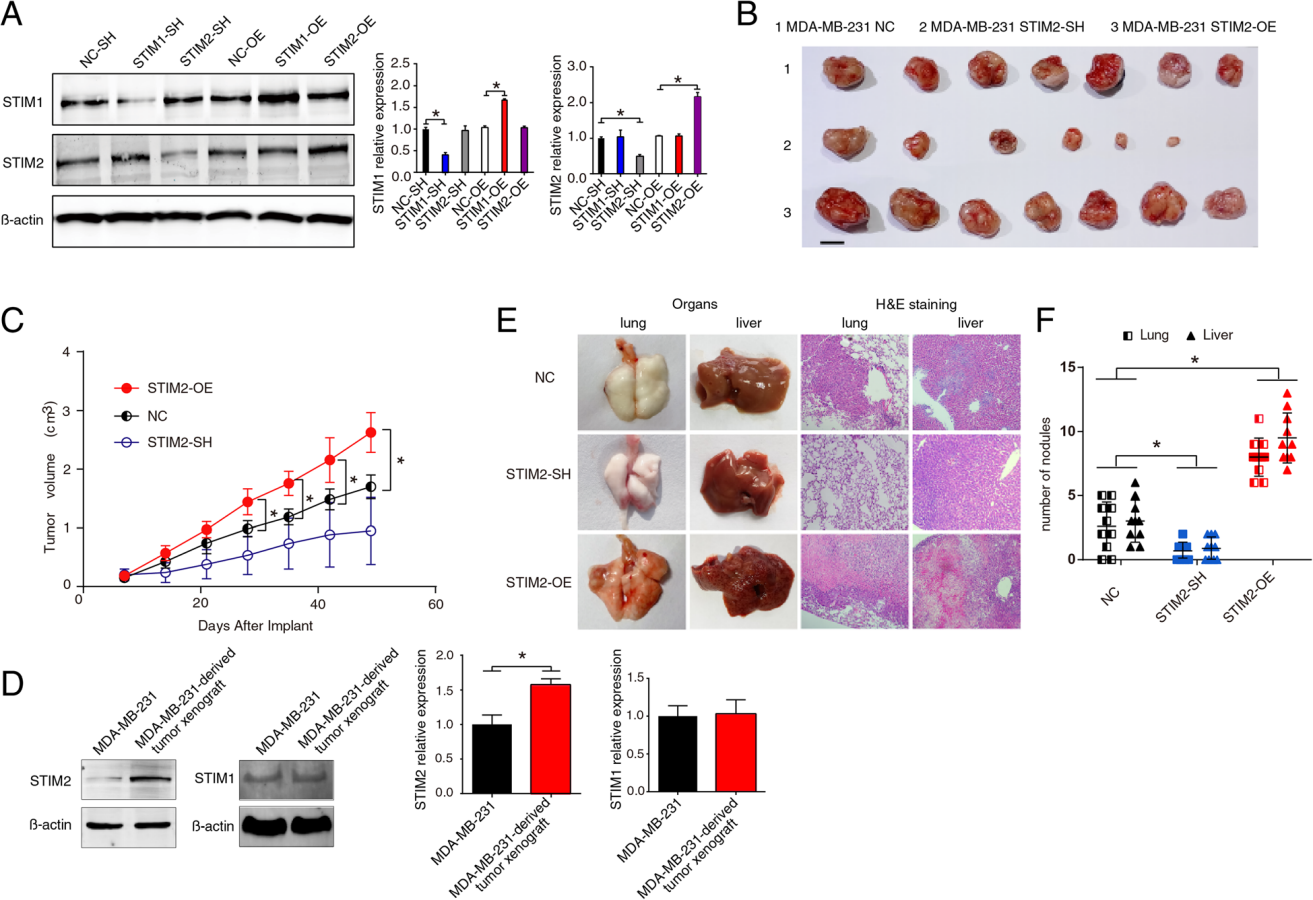
In vivo effects of STIM2 gain or loss on tumor growth and metastasis. **a** Western blots (left) and quantification of protein expression (right) showing knockdown (SH) or overexpression (OE) of STIM1 and STIM2 in MDA-MB-231 cells. **b** Effect of knockdown or overexpression of STIM2 on tumor growth. MDA-MB-231 cells transfected with lentiviruses were injected subcutaneously into nude mice. The mice were euthanized and tumors harvested 48 days after cancer cell injection. Photographs of representative tumors are shown. **c** Effect of knockdown or overexpression of STIM2 on tumor volume. Tumor volume was monitored every 3 days from day 7 after cancer cell inoculation by measuring tumor length and width with a sliding caliper. **d** Western blots (left) and quantification (right) showing the expression of STIM2 and STIM1 in parental MDA-MB-231 cells and MDA-MB-231-derived tumor xenografts. **e** Bright-field images (left) and hematoxylin and eosin (H&E) staining (right) of the lungs and livers of mice injected via the tail vein with STIM2-SH, STIM2-OE, or control (NC) cells at day 42 after injection. **f** Comparison of the number of metastatic nodules in the lungs and livers of mice injected via the tail vein with STIM2-SH, STIM2-OE, or NC cells at day 42 after injection. *T* tests or one-way ANOVA were used to compare independent groups. Data are presented as means ± SD of at least 3 independent experiments. *P < 0.05

To uncover the function of STIM2, lentiviral vector tools were used to knock down or overexpress STIM2 or STIM1 in the breast cancer cell line MDA-MB-231. Western blotting, which repeated at least three times, showed that knockdown or overexpression of STIM1 did not influence STIM2 expression and vice versa (Fig. 1a).

Implantation of STIM2-SH (knockdown) cells induced significantly less tumor growth than did implantation of control cells, whereas implantation of STIM2-OE cells induced larger tumors than did control cells (Fig. 1b, c). Intriguingly, xenograft tumors in the control group had higher expression levels of STIM2 than did the parental MDA-MB-231 cells, although the expression levels of STIM1 were the same (Fig. 1d).

STIM2 knockdown significantly reduced the number of metastatic nodules in the lungs and liver (*P* < 0.05), whereas STIM2 overexpression markedly (*P* < 0.05) increased the number of metastatic nodules these organs (Fig. 1e). These results suggest that gain of STIM2 enhances the growth and metastasis of breast cancer cells, whereas loss of STIM2 reduces this capability.

### Higher STIM2 protein expression is associated with human breast cancer metastasis

**Fig. 2:**
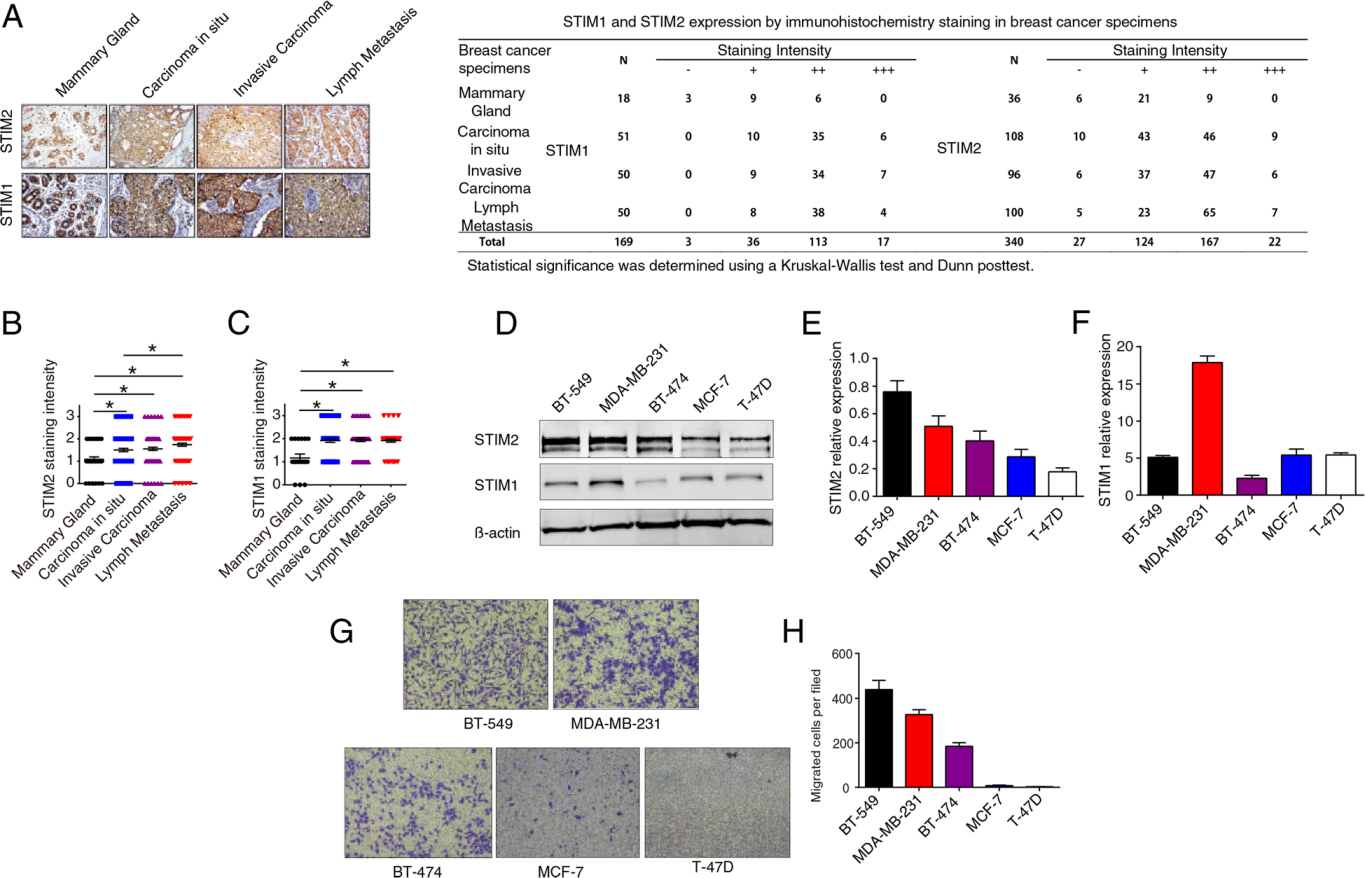
Higher STIM2 protein expression levels are associated with human breast cancer metastasis. **a** Representative images of immunohistochemical (IHC) staining (× 200) of STIM1 and STIM2 in mammary gland, carcinoma in situ, invasive carcinoma, and lymph node metastasis tissue microarrays. **b**, **c** Quantification of IHC staining intensity of STIM2 (**b**) and STIM1 (**c**) in breast cancer and normal mammary tissues. The staining intensity was scored with grades 0–3, and the data were analyzed with GraphPad Prism software version 6. Each symbol represents an individual sample. Statistical comparisons of staining intensity were performed using Kruskal-Wallis tests. **d**, **e**, **f** Representative Western blots (**d**) and quantification (**e**, **f**) of the protein expression levels of STIM1 and STIM2 in 5 breast cancer cell lines. **g**, **h** Images (× 20) (**g**) and quantification (**h**) of Transwell assays showing migration of 5 breast cancer cell lines. *T* tests or one-way ANOVA were used to compare independent groups. Data shown are means ± SD of at least 3 independent experiments. *P < 0.05

Protein expression was analyzed with IHC in 340 microarray samples for STIM2 and 169 samples for STIM1. The nuclear and cytoplasmic staining intensity of STIM2 was significantly stronger in breast cancer samples than in normal mammary epithelial tissue samples (Fig. 2a–c). Intriguingly, STIM2 protein level—evaluated as a combination of IHC staining intensity and the percentage of positively stained cells—was significantly higher in samples of breast cancer lymph node metastases than in primary breast tumor samples (Fig. 2b). We found a similar pattern for STIM1; in addition, no differences in STIM1 expression between ductal carcinoma in situ and invasive carcinoma were found (Fig. 2c).

We also examined the expression of STIM2 and STIM1 in a panel of breast cancer cell lines and found a higher level of STIM2 in two mesenchymal-like cell lines (MDA-MB-231, BT-549) than in three epithelial-like cell lines (T-47D, BT-474, and MCF7) (Fig. 2d–f). Transwell assays showed that the STIM2-high cells, including MDA-MB-231 and BT-549, migrated more than did the cells with lower STIM2 expression (Fig. 2g–h).

### Gain or loss of STIM2 affects EMT in breast cancer cells

**Fig. 3:**
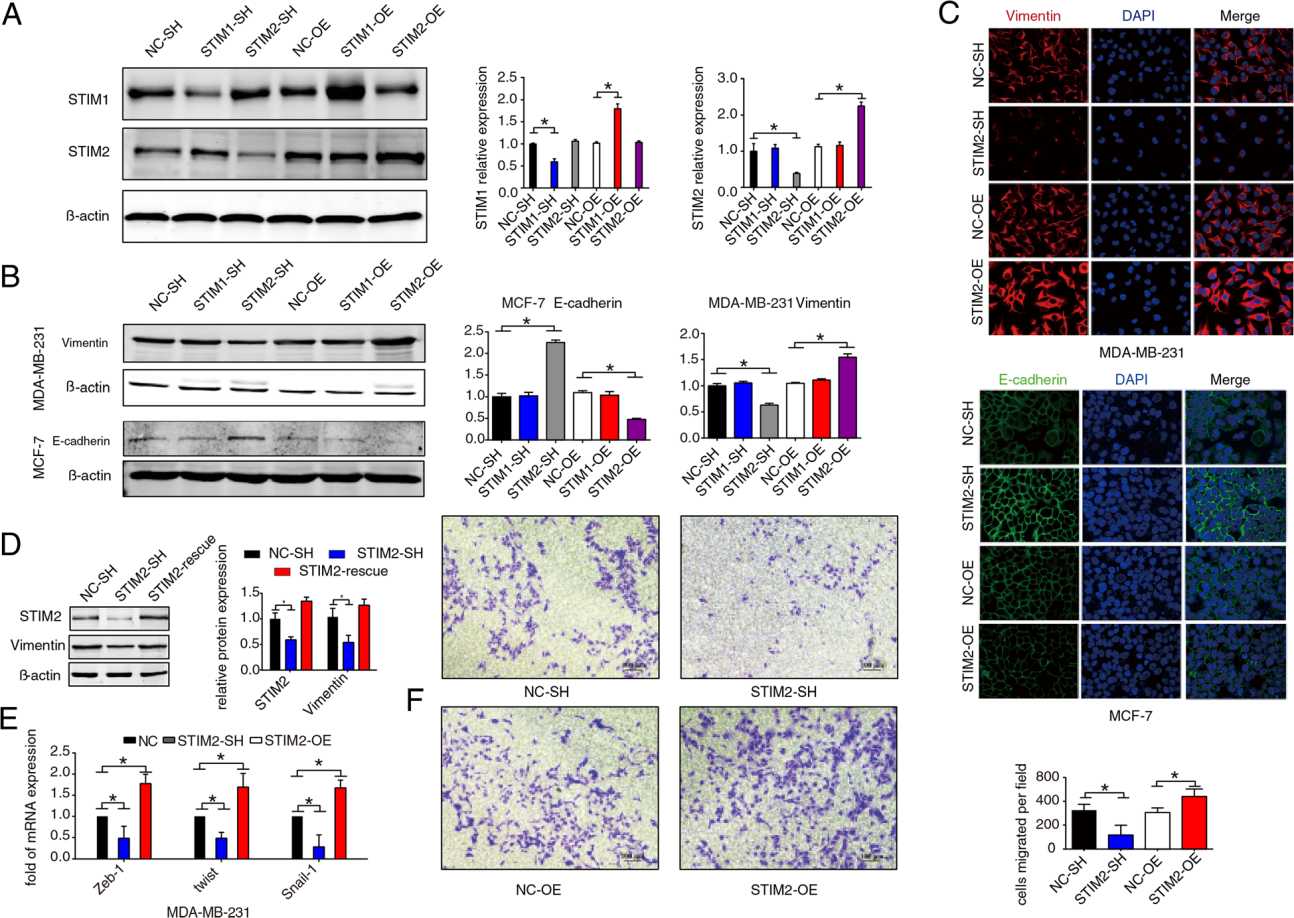
STIM2 regulates EMT in breast cancer cells. **a** Western blots (left) and quantification (right) showing knockdown (SH) or overexpression (OE) of STIM1 or STIM2 in MCF7 cells transfected with lentivirus. NC, control. **b** Western blots (left) and quantification (right) showing expression levels of the epithelial-mesenchymal transition (EMT) marker proteins E-cadherin in MCF7 cells and vimentin in MDA-MB-231 cells after knockdown or overexpression of STIM1 or STIM2. **c** Representative immunofluorescence images showing E-cadherin in MCF7 and vimentin in MDA-MB-231 cells with knockdown or overexpression of STIM2. **d** Western blots (left) and quantification (right) showing repression (rescue) of STIM2 in MDA-MB-231 cells with STIM2 knockdown and the impact of STIM2 repression on expression of vimentin. **e** mRNA expression of ZEB1, TWIST1, and SNAI1 were measured by qRT-PCR in MDA-MB-231 cells with STIM2 knockdown or overexpression. **f** Photomicrographs (left) and quantification (right) of Transwell assays showing migration of MDA-MB-231 cells with knockdown or overexpression of STIM2. *T* tests or one-way ANOVA were used to compare independent groups. Data shown are means ± SD from at least 3 independent experiments. *P < 0.05

Although STIM1 has been reported to affect focal adhesion turnover and rearrangement in several types of cancer cells [19], the role of STIM2 in regulating EMT and metastasis in breast cancer is unknown. Because we found an association between elevated STIM2 expression and enhanced cellular migration (Fig. 2d–f), we chose an epithelial-like breast cancer cell line with low metastatic potential, MCF7, for further comparison with the metastatic MDA-MB-231 cell line. Both cell lines were infected with lentivirus to knockdown STIM2 and STIM1 (STIM2-SH and STIM1-SH) or overexpress them (STIM2-OE and STIM1-OE). Western blotting confirmed that knockdown or overexpression of STIM1 did not influence STIM2 expression and vice versa (Fig. 3a).

We then investigated whether STIM2 influences EMT in breast cancer cells by determining expression levels of two markers of EMT, E-cadherin and vimentin. Knockdown of STIM2 significantly increased E-cadherin levels (*P* < 0.05) in MCF7 cell line but reduced vimentin levels (*P* < 0.05) in MDA-MB-231 cell line, whereas overexpression of STIM2 significantly reduced E-cadherin levels (*P* < 0.05) in MCF7 but markedly increased vimentin levels (*P* < 0.05) in MDA-MB-231. However, knockdown or overexpression of STIM1 did not affect E-cadherin and vimentin expression (Fig. 3b); these Western blotting results were corroborated by confocal immunofluorescence microscopy using double labeling for E-cadherin and vimentin (Fig. 3c).

To ensure that STIM2 regulation of EMT was not due to an off-target effect of lentiviral transduction, we reexpressed STIM2 in MDA-MB-231 cells that already had knockdown of STIM2. Reexpression of STIM2 rescued E-cadherin and vimentin to basal levels (Fig. 3d). Transcription of SNAI1, ZEB1, TWIST1, SMAD4, and SMAD5, which are related to EMT, was suppressed or enhanced when STIM2 was knocked down or overexpressed, respectively (Fig. 3e). In addition, silencing of STIM2 in breast cancer cells inhibited, whereas overexpression of STIM2 enhanced, the migration of breast cancer cells (Fig. 3f). Taken together, these results suggest that loss of STIM2 inhibits EMT and gain of STIM2 enhances EMT in breast cancer cells. Because knockdown or overexpression of STIM1 did not affect the expression of EMT markers in breast cancer cells, we conclude that STIM2, but not STIM1, regulates EMT in breast cancer cells.

### STIM2 specifically promotes the expression and nuclear translocation of transcription factor NFAT1

**Fig. 4:**
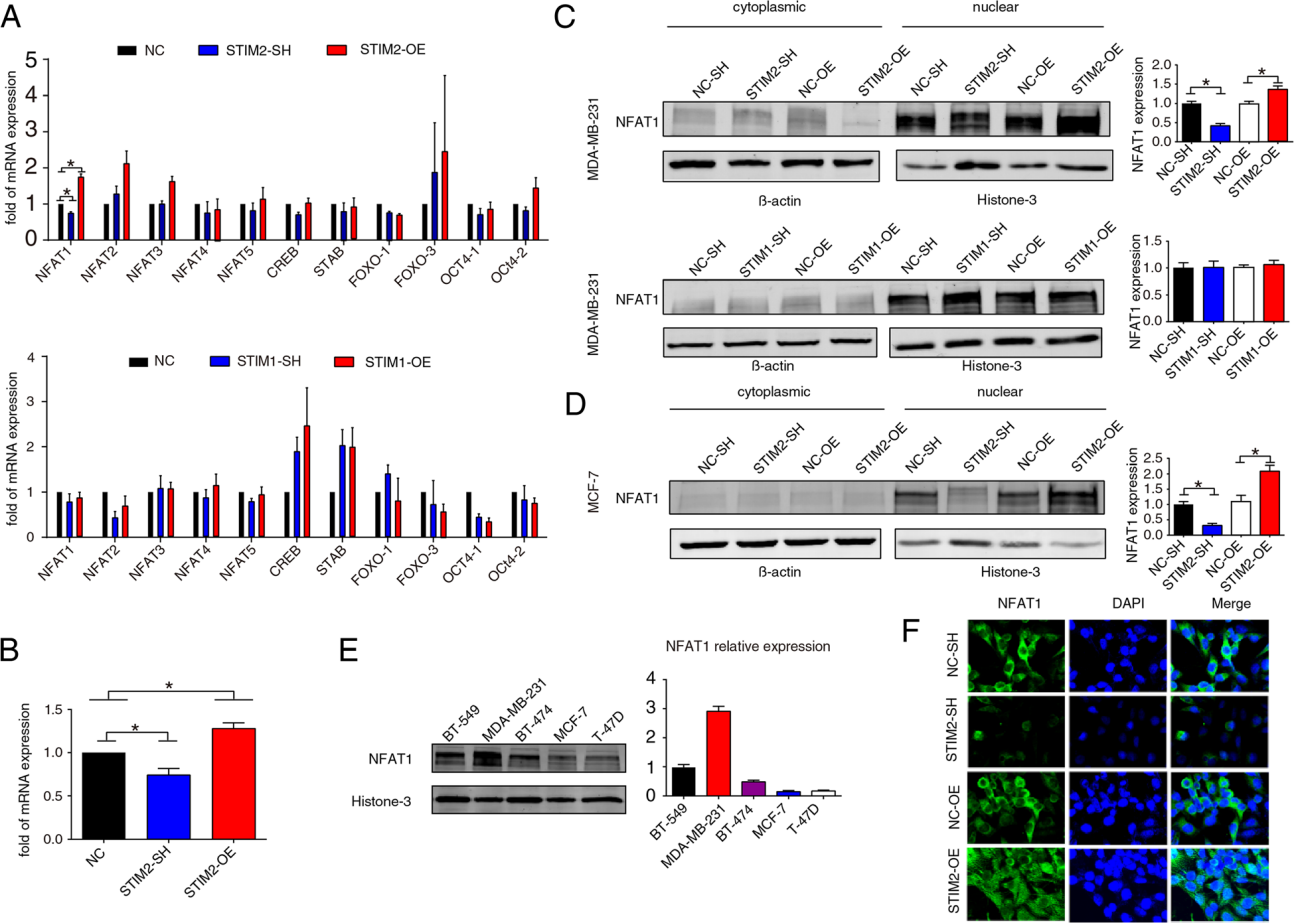
STIM2 regulates the expression and nuclear translocation of transcription factor NFAT1. **a** qRT-PCR analysis of the expression of transcription factors in MDA-MB-231 cells with STIM2 (top) or STIM1 (bottom) knockdown (SH) or overexpression (OE). **b** qRT-PCR analysis of the expression of NFAT1 in MCF7 cells with STIM2 knockdown or overexpression. **c** Western blots (left) and quantification (right) of NFAT1 protein levels in MDA-MB-231 cells with STIM2 or STIM1 knockdown or overexpression. **d** Western blots (left) and quantification (right) of NFAT1 protein levels in MCF7 cells with STIM2 knockdown or overexpression. **e** Western blots (left) and quantification (right) showing NFAT1 levels in the nuclear extracts of a panel of breast cancer cell lines. **f** Immunofluorescent staining (× 100) of NFAT1 in MDA-MB-231 cells with STIM2 knockdown or overexpression. One-way ANOVA was used to compare independent groups. Data shown are means ± SD of at least 3 independent experiments. *P < 0.05

It has been well documented that STIM2 regulates Ca^2+^. Thus, we hypothesized that STIM2 regulates EMT in breast cancer cells by influencing the intracellular Ca^2+^ level. Based on the assumption that knockdown or overexpression of STIM2 causes functional alterations of Ca^2+^-related transcription factors, we employed qRT-PCR to determine the expression of such transcription factors. Among the 11 transcription factors examined, NFAT1 was the only one with significant alteration following STIM2 transfection in MDA-MB-231. This alteration was found upon STIM2, but not STIM1, overexpression (Fig. 4a). The qRT-PCR analysis in MCF7 cells and Western blotting in MCF7 and MDA-MB-231 corroborated these results (Fig. 4b–d). Moreover, NFAT1 expression was significantly higher in the mesenchymal-like breast cancer cell lines with a higher migratory capacity (MDA-MB-231 and BT-549) (shown in Fig. 1d) than in the epithelial-like cell lines with a lower migratory capacity (BT-474, T-47D, and MCF7) (Fig. 4e). Finally, STIM2 regulated not only the expression of NFAT1 but also its nuclear translocation, as shown by the confocal immunofluorescence microscopy imaging (Fig. 4f). These results support the conclusion that NFAT1 is a downstream target of STIM2.

### NFAT1 promotes EMT in breast cancer cells

**Fig. 5:**
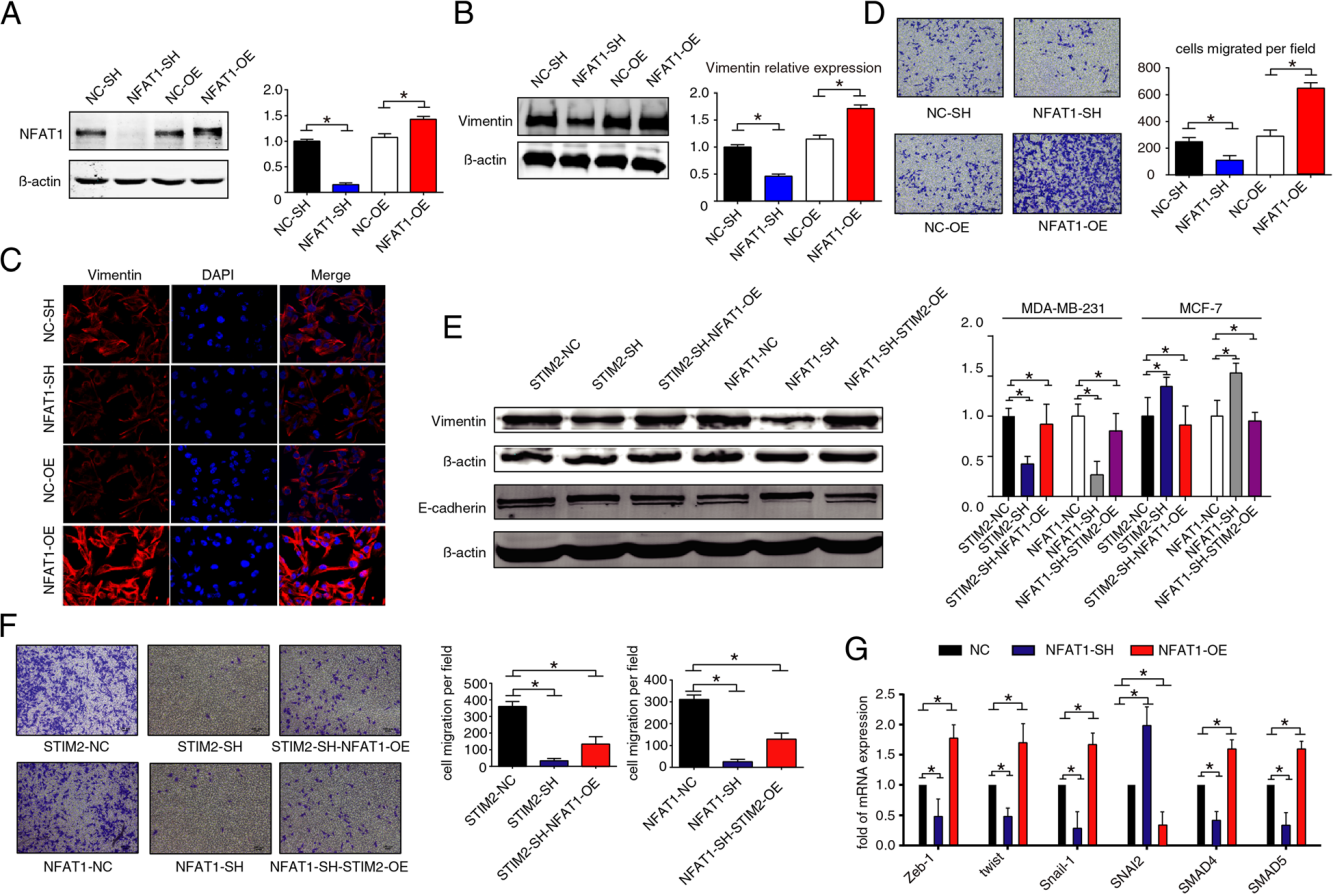
NFAT1 regulates EMT in breast cancer cells. **a** Western blots (left) and quantification (right) showing knockdown (SH) or overexpression (OE) of NFAT1 in MDA-MB-231 cells. **b** Western blots (left) and quantification (right) of expression of the EMT marker proteins vimentin in MDA-MB-231 cells with knockdown or overexpression of NFAT1. **c** Confocal images of immunofluorescence staining (× 100) of vimentin in MDA-MB-231 cells with knockdown or overexpression of NFAT1. **d** Images (right) and quantification (left) of Transwell assay showing migration of MDA-MB-231 cells with NFAT1 knockdown or overexpression. *T* tests or one-way ANOVA were used to compare independent groups. Data shown are means ± SD of at least 3 independent experiments. *P < 0.05. NC, control. **e** Western blots (left) and quantification (right) showing rescue of vimentin and E-cadherin expression in MDA-MB-231 and MCF7 cells with STIM2 knockdown (SH) and/or overexpression (OE) of NFAT1. **f** Images and quantification of transwell assays showing migration of MDA-MB-231 cells with STIM2-SH/NFAT1-OE and MDA-MB-231 cells with NFAT1-SH/STIM2-OE. **g** Effects of knockdown or overexpression of NFAT1 on the transcription of ZEB1, TWIST1, SNAI1, SNAI2, SMAD4, and SMAD5, in MDA-MB-231 cells. *T* tests or one-way ANOVA were used to compare independent groups. Data shown are means ± SD of at least 3 independent experiments. *P < 0.05. NC, control

To investigate the role of NFAT1 in regulating EMT, MDA-MB-231 cells were transfected with a lentivirus to knock down or overexpress NFAT1 (Fig. 5a). MDA-MB-231 cells with NFAT1 knockdown exhibited reduced vimentin expression, whereas NFAT1 overexpression was accompanied by significantly higher expression of vimentin (*P* < 0.05) (Fig. 5b). These results were corroborated by confocal immunofluorescence microscopy (Fig. 5c).

In addition, silencing of NFAT1 inhibited the migration of MDA-MB-231 cells, while overexpression of NFAT1 enhanced their migration (Fig. 5d). These results suggest that NFAT1 specifically regulates EMT and the motility of breast cancer cells.

To further confirm the relationship of STIM2 and NFAT1, we overexpressed NFAT1 in STIM2-SH MDA-MB-231 and MCF7 cells. In STIM2-knockdown cells overexpressing NFAT1, expression of vimentin and E-cadherin returned to basal levels (Fig. 5e). Overexpression of NFAT1 enhanced the migration of STIM2-knockdown MDA-MB-231 cells, as shown by transwell assays (Fig. 5f). In addition, with knockdown of NFAT1 in MDA-MB-231 cells, the transcription of SNAI2, a repressor of the Snail family, was enhanced, whereas the transcription of SNAI1, ZEB1, TWIST1, SMAD4, and SMAD5 was suppressed. As expected, overexpression of NFAT1 led to the opposite outcome (Fig. 5g).

### NFAT1 upregulates TGF-β1 expression and secretion

**Fig. 6:**
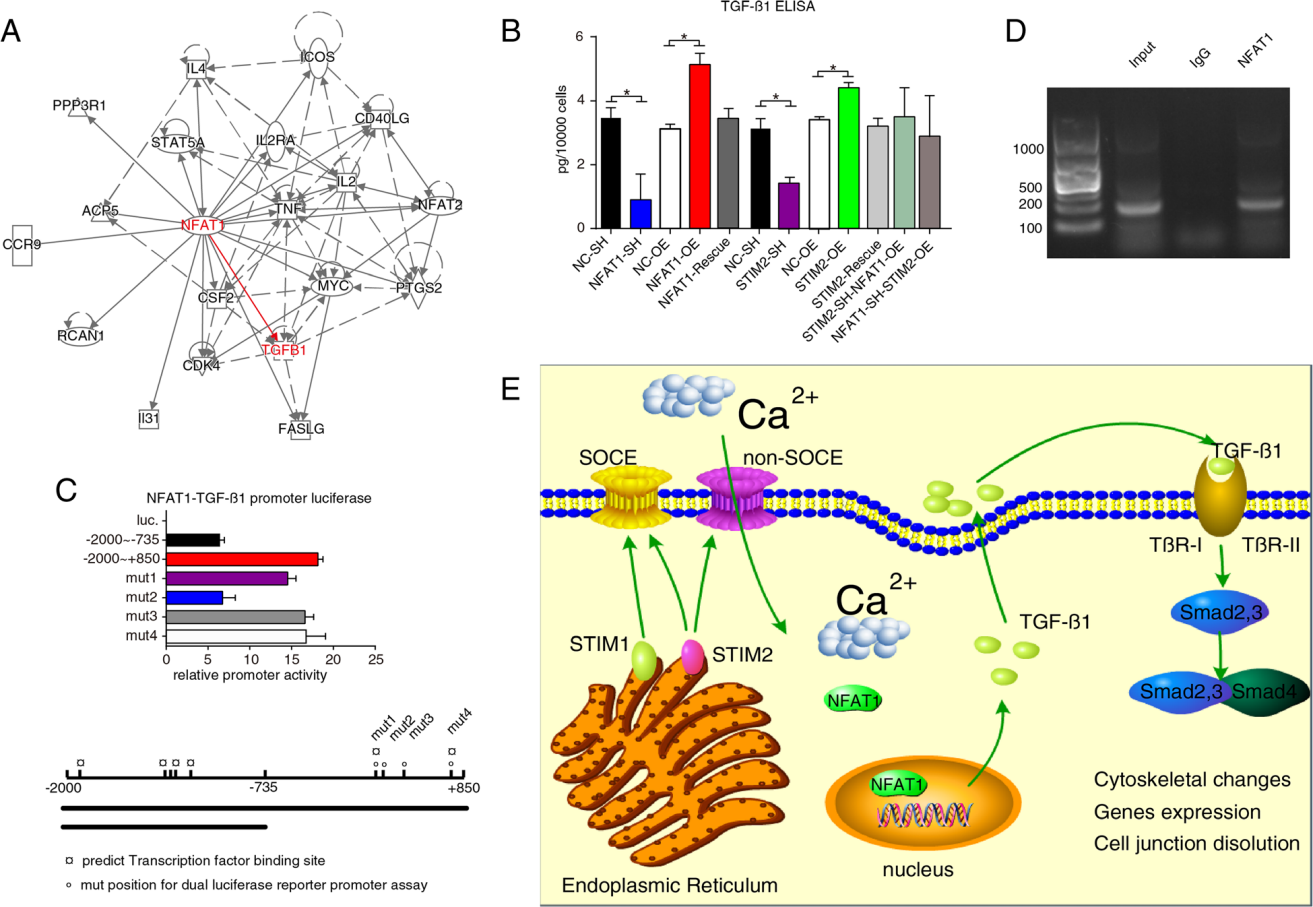
Effects of NFAT1 on TGF-β1 expression and secretion. **a** Ingenuity Pathway Analysis summary. Using IPA, we identified downstream molecules of NFAT1, including TGF-β1, a key regulator of EMT. **b** Results of ELISA showing the secretion of TGF-β1 with knockdown (SH), overexpression (OE), or rescue of NFAT1 and STIM2. **c** Results of dual-reporter luciferase assay showing NFAT1-dependent activation of the TGF-β1 promoter. Reporter gene constructs were transfected into HEK 293 T cells, and luciferase activity was measured after 48 h. **d** Chromatin immunoprecipitation of NFAT1 on the TGF-β1 promoter. After cells were lysed, proteins were cross-linked and immunoprecipitated with an anti-NFAT1 antibody. The cross-linking was reversed, and PCR was performed. One of 3 separate experiments is shown. **e** Signaling pathway formed by STIM2, NFAT1, and TGF-β1 was showed. *T* tests or one-way ANOVA were used to compare independent groups. Data shown are means ± SD from at least 3 independent experiments. NC, control. *P < 0.05

We next performed IPA to identify the genes that are regulated by NFAT1 and to identify the EMT-regulating genes among the NFAT1-regulated genes. Correlation of NFAT1 and TGF-β1 was reported in tolerant T cells [20]. We further mined IPA database and found association network for NFAT1 and TGF-β1 (Fig. 6a). TGF-β family proteins are key inducers of EMT [21, 22]. In particular, TGF-β1 induces EMT in wound healing, fibrosis, and cancer [23], leading to the speculation that NFAT1 might regulate TGF-β1 in immune cells [24]. To validate the interactions and correlations among STIM2, NFAT1, and TGF-β1 shown in Figs. 4, 5, and 6a, we performed ELISA assays. As shown in Fig. 6b, silencing of NFAT1 significantly reduced the amount of TGF-β1 in culture medium (*P* < 0.05), whereas overexpression of NFAT1 significantly increased the secretion of TGF-β1. Intriguingly, rescue-expression of NFAT1 in the NFAT1-SH cells recovered TGF-β1 level to the basal level in culture medium, comparable to that of control cells (Fig. 6b). Similarly, knockdown of STIM2 reduced the secretion of TGF-β1, whereas overexpression of STIM2 increased TGF-β1 secretion (Fig. 6b).

In Fig. 5, we present results that support STIM2 to be upstream of NFAT1 in regulating TGF-β1 secretion and EMT. We also noticed vimentin expression in rescue experiment (Fig. 5e), indicating that even rescue of STIM2 expression in the absence of NFAT1 also recovers vimentin expression to some degree which hints towards involvement of NFAT1-independent mechanism of STIM2. We therefore measured TGF-β1 secretion using the same rescue conditions in STIM2-SH-NFAT1-OE and NFAT1-SH-STIM2-OE cells (Fig. 6b). As expected, TGF-β1 secretion was increased in both conditions comparable to that of control cells, in line with the results seen in Fig. 5e.

Next, we determined whether NFAT1 regulates TGF-β1 at the transcriptional level. We cloned human TGF-β1 promoter region covering − 2000 to + 850 and − 2000 to − 735 fragments. Deletion of the fragment from − 735 to + 850 resulted in a reduction of luciferase activity of about 70%. To determine the binding site of NFAT1 involved in regulating the expression of TGF-β1, we used the JASPAR database of transcription factor binding profiles (http://jaspar.genereg.net/) to generate stepwise deletion constructs extending from − 2000 to + 850 with distinct point mutations. The construct containing the mutation from + 305 to + 344 showed the lowest reporter activity (Fig. 6c). Furthermore, ChIP assays revealed binding of NFAT1 in this TGF-β1 promoter region in MDA-MB-231 cells (Fig. 6d). Thus, these results suggest that the proximal promoter region from + 305 to + 344 plays a dominant role in NFAT1 regulation of the synthesis and secretion of TGF-β1.

## Discussion

Our results demonstrate for the first time that STIM2 is a master regulator of NFAT1 in breast cancer. STIM2 governs the transcription and nuclear retention of NFAT1 to regulate TGF-β1 and associated signaling. Thus, we have uncovered a novel mechanism by which STIM2 distinctively modulates EMT to promote breast cancer cell motility and tumor metastasis. Our findings suggest that STIM1 and STIM2 differentially regulate intracellular Ca^2+^ via different downstream signaling pathways and indicate that STIM2 plays an important but previously unappreciated role in promoting breast cancer metastasis.

This study reveals several new aspects of the role of STIM family members, particularly STIM2, in EMT and breast cancer metastasis. First, our study revealed for the first time that STIM2 is involved in EMT via NFAT1 and TGF-β1 signaling in human metastatic breast cancer cells. Previously, this phenomenon has only been reported in mouse immune cells [20]. Second, we found that STIM2, but not STIM1, promotes EMT in metastatic breast cancer cells. Thus, our study delineates a novel connection between STIM2 and EMT, via bridging of NFAT1 and TGF-β1. Third, we have demonstrated for the first time that NFAT1 regulates the transcription of TGF-β1 in breast cancer cells.

When Ca^2+^ in the ER is depleted, STIMs are the major components of store-operated Ca^2+^ channels, by gating and opening Orai channels in the plasma membrane [11]. Despite the notable difference in the functions of STIM1 and STIM2, their C-terminal regions are highly conserved [24]. STIM2 is more sensitive to minor changes in ER Ca^2+^ concentration than STIM1. This greater sensitivity for free Ca^2+^ allows STIM2 to react to ER Ca^2+^ concentration fluctuations and activate earlier than STIM1 upon agonist-induced ER Ca^2+^ store discharge [18]. Therefore, STIM2 and STIM1 may regulate different signaling pathways (SOCE and non-SOCE), with STIM2 playing a more important role in regulating breast cancer cell motility and promoting metastasis.

Both STIMs and Orai proteins, recently identified as critical constituents of SOCE, are implicated in cancer cell motility and metastasis [13, 25, 26]. Therefore, we focused on specific Ca^2+^-dependent transcription factors; NFAT1 drew our attention. Originally characterized in immune cells, NFAT factors act as Ca^2+^-dependent transcription factors promoting the transcription of key genes in the development and activation of lymphocytes and differentiation of cardiac muscle cells [27, 28]. NFAT1 is known to contribute to the migration of cancer cells [29–37], including breast cancer cells [38, 39]. Thus, our results are in line with the existing literature.

In IPA, the only report [20] describing the relationship between NFAT1 and TGF-β1, which is a key regulator of EMT in cancers [21, 40, 41], focused on tolerant T cells and did not explore this relationship in cancer cells. In the current study, we provided evidence showing the activation of an NFAT1–TGF-β1 axis in breast cancer cells along with its upstream driver STIM2. Moreover, we showed that the overexpression of NFAT1 in breast cancer cells leads to enhanced EMT in these cells, whereas NFAT knockdown leads to reduced secretion of TGF-β1. These findings highlight the roles and importance of STIM2 and NFAT1 in breast cancer tumor progression and metastasis.

Based on our observation in Fig. 5e, we speculate potential involvement of NFAT1-independent mechanisms by which STIM2 regulates vimentin expression. As TGF-β1 plays a critical role in regulating a broad range of cellular functions and physiological processes, there could be other mechanisms that regulate TGF-β1 expression and secretion [42–44]. Meanwhile, same conditions (cells with STIM2-SH-NFAT1-OE and NFAT1-SH-STIM2-OE) were used to evaluate the release of TGF-β1 in rescue experiments, and TGF-β1 secretion was recovered to control cell level as shown in Fig. 6b, suggesting the STIM2-NFAT1-TGF-β1 is at least one of the important regulatory pathways and potential involvement of other pathways (Fig. 6e).

It has been documented that STIM2 can regulate Ca^2+^-dependent transcription factor NFAT1 by impacting SOCE or non-SOCE, and promoting the expression and the translocation of NFAT1 enhances the secretion of TGF-β1. TGF-β1 which regulates cellular functions and plays a key role in development and carcinogenesis, by activating its intracellular effectors and forming trimetric complexes with Smad proteins including Smad2 and Smad3 (R-Smad) via Tβ-receptors I and II and Smad4 (Co-Smad), facilitating translocation into the nucleus and subsequent transcription of relevant genes. In addition, TGF-β receptors activate Smad-independent pathways that not only regulate Smad signaling, but also allow Smad-independent TGF-β responses [45–48]. Our results suggest that the STIM2-NFAT1-TGF-β1 pathway can regulate EMT and migration of breast cancer cells (Fig. 6e).

Although both STIM1 and STIM2 sense calcium release, leading to influx of extracellular calcium [49–51], our results demonstrate that gain or loss of STIM1 alone has minimal effect on EMT, whereas gain or loss of STIM2 has a substantial impact on EMT in breast cancer cells. Our results also demonstrate that STIM2 influences not only exogenous TGF-β-induced EMT, but also the expression and translocation of NFAT1, which further regulates the synthesis and secretion of endogenous TGF-β1 (Fig. 6e). While we previously showed that STIM1 specifically influences *exogenous* TGF-β-induced EMT [52], results in this work further our understanding of the biology of STIM family members in cancer cells and provide a rationale for potential precise targeting for cancer therapy.

## Conclusions

In summary, the present study demonstrates the importance of a newly described signaling pathway formed by STIM2, NFAT1, and TGF-β1. This pathway promotes metastasis by inducing EMT and motility of breast cancer cells. As the central node of this axis, STIM2 activation may serve not only as a prognostic factor to predict clinical outcomes for breast cancer patients, but also as a potential therapeutic target (Fig. 6e).

## Additional files

Additional file 1: Online Supplementary Methods Section. (DOCX 20 kb)

Additional file 2: IACUC Aprroval Document for Mouse Breast Cancer Xenograft Tumor Assessments. (PDF 643 kb)

Additional file 3: IRB Approval Document for Human Breast Cancer Tissue Microarray Assessments. (PDF 2603 kb)

## Data Availability

The datasets used and/or analyzed in this study are available from the corresponding author on reasonable request.

## Abbreviations

STIM2: Stromal interaction molecule 2
SOCE: Store-operated Ca^2+^ entry
EMT: Epithelial-mesenchymal transition
TGF-β1: Transforming growth factor-β
NFAT1: Nuclear factor of activated T cells 1
