# Supplementary material for: Calcium-sensing stromal interaction molecule 2 upregulates nuclear factor of activated T cells 1 and transforming growth factor-β signaling to promote breast cancer metastasis
Source: Breast Cancer Res. 2019 Aug 29;21:99. doi: 10.1186/s13058-019-1185-1 (PMC6716836; doi:10.1186/s13058-019-1185-1)
Supplement: Supplementary file 1 — Online Supplementary Methods Section. (DOCX 20 kb) [file 13058_2019_1185_MOESM1_ESM.docx]

**STIM2 Regulates NFAT1/TGF-β1 Pathway to Potentiate EMT and Promote Breast Cancer Metastasis**

Yutian Miao^1^, Qiang Shen^2^, Siheng Zhang^1^, Hehai Huang^1^, Xiaojing Meng^1^, Xianchong Zheng^1^,

Zhuocheng Yao^1^, Zhanxin He^1^, Sitong Lu^1^, Chunqing Cai^1,*^, Fei Zou^1,*^

**On-line only methods**

**Lentiviral infection and generation of stable expression cell lines**

STIM1, STIM2, and NFAT1 stable knockdown (STIM1-SH, STIM2-SH, and NFAT1-SH) and overexpression (STIM1-OE, STIM2-OE, and NFAT1-OE) clones were generated using commercially packaged lentiviral vectors (Hanbio, Shanghai, China and Genechem, Shanghai, China). Control clones were established using the empty lentiviral vector. Lentiviral infection and selection of stable expression clones were performed according to the manufacturer’s instructions. Briefly, purified lentiviruses were infected into the cells with a prior incubation with 5µg/ml polybrene for 30 min at 37°C. After virus infection for 24 h, the culture medium containing lentivirus was replaced with fresh medium and supplemented with 1µg/ml puromycin 48 h later for 24 h. Knockdown and overexpression of the genes were determined to be stable for at least 6 passages in culture by Western blot.

**Transwell migration assay**

The effects of gain/loss of STIM1, STIM2, or NFAT1 on the migration of breast cancer cells were determined using 24-well transwell plates with an 8-µm pore size (Costar, USA) *in vitro*. The bottom chamber was filled with culture medium containing 10% FBS, and 5 × 10^4^of cells suspended in serum-free medium were placed on the inserts in the upper chambers for culture at 37^0^C in 5% CO_2_ . After incubation for designated time (12 h for MDA-MB-231), the cells on the upper filter surface were removed using a cotton swab. Cells penetrating and attaching to the lower filter surface were fixed with 4% formaldehyde and stained with 0.5% crystal violet. Images of the stained cells were acquired under a 20 × objective. Migrated cells were quantified by Image Pro Plus 6.0 software (Media Cybernetics, U.S.A.).

**RNA extraction and Real-time Quantitative PCR (****qRT-PCR)**

Total RNA was extracted from the cultured cells using RNAiso Plus (Takara, Japan) and 1 µg aliquots of total RNA was subjected to synthesize cDNA with a PrimeScript™RT reagent Kit (Takara) according to the product manual. A quantitative analysis of indicated mRNA expression was determined by qRT-PCR using a SYBR^®^ Premix Ex Taq^TM^ kit (Takara). The qRT-PCR was performed on LightCycler^®^ 96 Real-Time PCR System (Roche, USA) for 40 cycles and was repeated in at least three independent experiments using different RNA samples. Expression levels were calculated relative to those of the mRNA of the housekeeping GAPDH using the 2^-ΔΔCt^ method. The sequences of primers used for amplification were listed in Supplemented Materials.

**Western blot analysis**

Cells were lyzed in RIPA lysis buffer (KeyGEN, China). A total of 40 µg of denatured protein extracts were resolved in 8–15% SDS-polyacrylamide gel electrophoresis and transferred to polyvinylidene difluoride membranes. Blocking was performed in 5% bovine serum albumin (BSA) dissolved in Tris buffered saline containing 0.1% Tween 20 (TBST) for 1h at room temperature. PVDF membrane was then incubated with specific primary antibodies (STIM1, 1:1,000, A7411, Abclone; STIM2, 1:1,000, PRS4125, Sigma; NFAT1, 1:1,000, 22023-1-AP, Proteintech; E-cadherin, 1:1,000, 14472S, CST; vimentin, 1:1,000, 5741T, CST; histone3, 1:5,000, 17168-1-AP, Proteintech; and β-actin, 1:15,000, RM2001, RAY Antibody), diluted in TBST containing 3% BSA overnight at 4°C. Peroxidase-conjugated secondary antibodies against mouse (1:15,000, A0168, Sigma), rabbit (1:15,000, A0545, Sigma) were used. Signal was detected by using a Li-COR odyssey infrared imaging system (LI-COR Biosciences).

**Tissue microarray and immunohistochemistry (IHC)**

Human breast cancer tissue microarrays were obtained from US Biomax (BR8011 and BR10010d) for IHC as paraffin embedded sections. For further analysis, missing or inconclusive cores were removed, and the remaining 595 unique breast cancer cases and 75 normal breast cases were included for study. An attending pathologist confirmed histological diagnosis of breast cancer for the tissue arrays. For IHC staining, sections were incubated with STIM1 (1:100, A7411, Abclone) or STIM2 (1:100, PRS4125, Sigma) antibodies overnight at 4°C after deparaffinization, rehydration, antigen retrieval, quenching of endogenous peroxidase and blocking. Reactions were developed through EliVisionTMplus kit (MXB, China) and signal was visualized using DAB visualization kit (MXB, China) according to the manufacturer’s instructions. IHC staining scores of intensity and density of positive cells were defined by experienced pathology doctors from Ailina Biotechnology Company (Xian, China, www.ailenabio.com).

**Tumor** **xenografts and *in vivo* metastasis**

Six-week-old female BALB/c-nu mice were obtained and maintained under defined conditions at the Laboratory Animal Center of Southern Medical University (Guangzhou, China). Procedures involving animals and their care were in accordance with the legal mandates and national guidelines. Moreover, Southern Medical University Experimental Animal Ethics Committee approved the experimental protocol (Authorization No. 2016038). STIM1 and STIM2 stable knockdown or overexpression clones, as well as control clones developed inMDA-MB-231 cells were used in xenografts and *in vivo* metastasis assays. For xenograft tumor assay, cells (5×10^6^) were resuspended in 100 µl of PBS and were inoculated into the mammary fat pad of mice (n=7 per group). Tumor growth was evaluated by monitoring tumor volume (TV = length × width^2^ × 0.5) every 3 days for 8 weeks. For *in vivo* metastasis assay, cells (2.5 × 10^5^) were injected via tail vein and the mice were maintained for 6 weeks. The mice were sacrificed at specific time points; and their tumor xenografts, the lungs and livers were harvested for further evaluation. Paraffin sections (4µm) of harvested lungs and livers were stained with hematoxylin and eosin (H&E) and images were acquired under a 20 × objective.

**Immunofluorescence (IF)**

Cells were grown in a confocal dish (NEST, China) and allowed to attach for 24 h. Cells were fixed in 4% paraformaldehyde for 20 min at RT and penetrated using precooled methanol for 10 min at -20°C. After blocking with normal goat serum for 30 min, cells were incubated with E-cadherin (1:200, 14472S, CST), vimentin (1:200, 5741T, CST) overnight at 4°C. Cells were then washed with phosphate-buffered saline (PBS) and incubated with goat anti-rabbit and goat anti-mouse secondary antibody (Invitrogen, CA, USA) for 1 h in the dark at RT. Nucleus were stained using DAPI (KeyGEN, China). Images were acquired under a ×60 oil objective on an Olympus FV1000 laser scanning confocal microscope (Olympus, FV1000-IX71, Japan).

**Chromatin immunoprecipitation assay (ChIP)**

The Chromatin immunoprecipitation assay (ChIP) was performed using the ChIP-IT Express Kit (Active Motif, Shanghai, China) according to the manufacturer’s protocol. Specific PCR was performed by using the following primers that can identify NFAT1 binding sites: 5′-CAGCCTGAGGCCCCAGAGTC-3′; and 5′-TCCCCAAGTCCTGCCTCCTC-3′. The PCR products were run on a 1% agarose gel.

## Sequences of primers used for amplification

| NFAT1-F | CACCGCATCACAGGGAAGAC |
| --- | --- |
| NFAT1-R | GCACAGTCAATGACGGCTC |
| NFAT2-F | GAGCCGAATGCACATAAGGTC |
| NFAT2-R | CCAGAGAGACTAGCAAGGGG |
| NFAT3-F | TCCACCTCCATCTACTTTAACCA |
| NFAT3-R | TTGGGACCACCTAATGGGCT |
| NFAT4-F | CTTCTCCGATGCCTCTGACG |
| NFAT4-R | CGGGGCTTGGACCATACAG |
| NFAT5-F | GCTGGATAACAGTCGGATGTC |
| NFAT5-R | GCCTCTGCTTTGGATTTCGTT |
| SATB1-F | AGCAGGAAATGAAGCGTGCTAAAG |
| SATB1-R | GATCATGGAGAGGTTCTCCCACAG |
| OCT4-1F | CCATGGCGGGACACCTCGCT |
| OCT4-1R | TGTGTTCCCAATTCCTTTCTTAGTG |
| OCT4-2F | AGCTCCTAAAGCAGAAGAGGATCAC |
| OCT4-2R | CCGCAGCTTACACATGTTCTTGAA |
| CREB-F | GCTGCCTCTGGAGACGTACAA |
| CREB-R | GCTAGTGGGTGCTGTGCGA |
| FOXO1-F | AAGAGCGTGCCCTACTTCAA |
| FOXO1-R | TTCCTTCATTCTGCACACGA |
| FOXO3-F | GGCTGGAAGAACTCTATC |
| FOXO3-R | GTACTTGTTGCTGTTGTC |
| GAPDH-F | CTCCTCCACCTTTGACGCTG |
| GAPDH-R | CATACCAGGAAATGAGCTTGACAA |

Four mutants（A-C,G-T）

1

AGTTTCCCTATCTGTAAATTGGGGACAGTAAATGTATGGGGTCGCAGGGTGTTGAGTGACAGGAGGCTGCTTAGCCACATGGGAGGTGCTCAGTAAAGGAGAGCAATTCTTACAGGTGTCTGCCTCCTGACCCTTCCATCCTTCAGGTGTCCTGTTGCCCCCTCCTCCCACTGACACCCTCCGGAGGCCCCCATGTTGACAGACCCTCTTCTCCTACCTTGTTTCCCAGCCTGACTCTCCTTCCGTTCTGGGTCCCCCTCCTCTGGTCGGCTCCCCTGTGTCTCATCCCCCGGATTAAGCCTTCTCCGCCTGGTCCTCTTTCTCTGGTGACCCACACCGCCCGCAAAGCCACAGCGCATCTGGATCACCCGCTTTGGTGGCGCTTGGCCGCCAGGAGGCAGCACCCTGTTTGCGGGGCGGAGCCGGGGTGCCCGCCCCCTTTCCCCCAGGGCTGAAGGGACCCCCCTCGGAGCCCGCCCACGCGAGATGAGGACGGTGGCCCAGCCCCCCCATGCCCTCCCCCTGGGGGCCGCCCCCGCTCCCGCCCCGTGCGCTTCCTGGGTGGGGCCGGGGGCGGCTTCAAAACCCCCTGCCGACCCAGCCGGTCCCCGCCGCCGCCGCCCTTCGCGCCCTGGGCCATCTCCCTCCCAGCCGGTCCCCGCCGCCGCCGCCCTTCGCGCCCTGGGCCATCTCCCTCCCACCTCCCTCCGCGGAGCAGCCAGACAGCGAGGGCCCCGGCCGGGGGCAGGGGGGACGCCCCGTCCGGGGCACCCCCCCGGCTCTGAGCCGCCCGCGGGGCCGGCCTCGGCCCGGAGCGGAGGAAGGAGTCGCCGAGGAGCAGCCTGAGGCCCCAGAGTCTGAGACGAGCCGCCGCCGCCCCCGCCACTGCGGGGAGGAGGGGGAGGAGGAGCGGGAGGAGGGACGAGCTGGTCGGGAGAAGAGGAAAA(CTTCCCC)AAACTTTTGAGACTTTTCCGTTGCCGCTGGGAGCCGGAGGCGCGGGGACCTCTTGGCGCGACGCTGCCCCGCGAGGAGGCAGGACTTGGGGACCCCAGACCGCCTCCCTTTGCCGCCGGGGACGCTTGCTCCCTCCCTGCCCCCTACACGGCGTCCCTCAGGCGCCCCCATTCCGGACCAGCCCTCGGGAGTCGCCGACCCGGCCTCCCGCAAAGACTTTTCCCCAGACCTCGGGCGCACCCCCTGCACGCCGCCTTCATCCCCGGCCTGTCTCCTGAGCCCCCGCGCATCCTAGACCCTTTCTCCTCCAGGAGACGGATCTCTCTCCGACCTGCCACAGATCCCCTATTCAAGACCACCCACCTTCTGGTACCAGATCGCGCCCATCTAGGTTATTTCCGTGGGATACTGAGACACCCCCGGTCCAAGCCTCCCCTCCACCACTGCGCCCTTCTCCCTGAGGACCTCAGCTTTCCCTCGAGGCCCTCCTACCTTTTGCCGGGAGACCCCCAGCCCCTGCAGGGGCGGGGCCTCCCCACCACACCAGCCCTG

2

AGTTTCCCTATCTGTAAATTGGGGACAGTAAATGTATGGGGTCGCAGGGTGTTGAGTGACAGGAGGCTGCTTAGCCACATGGGAGGTGCTCAGTAAAGGAGAGCAATTCTTACAGGTGTCTGCCTCCTGACCCTTCCATCCTTCAGGTGTCCTGTTGCCCCCTCCTCCCACTGACACCCTCCGGAGGCCCCCATGTTGACAGACCCTCTTCTCCTACCTTGTTTCCCAGCCTGACTCTCCTTCCGTTCTGGGTCCCCCTCCTCTGGTCGGCTCCCCTGTGTCTCATCCCCCGGATTAAGCCTTCTCCGCCTGGTCCTCTTTCTCTGGTGACCCACACCGCCCGCAAAGCCACAGCGCATCTGGATCACCCGCTTTGGTGGCGCTTGGCCGCCAGGAGGCAGCACCCTGTTTGCGGGGCGGAGCCGGGGTGCCCGCCCCCTTTCCCCCAGGGCTGAAGGGACCCCCCTCGGAGCCCGCCCACGCGAGATGAGGACGGTGGCCCAGCCCCCCCATGCCCTCCCCCTGGGGGCCGCCCCCGCTCCCGCCCCGTGCGCTTCCTGGGTGGGGCCGGGGGCGGCTTCAAAACCCCCTGCCGACCCAGCCGGTCCCCGCCGCCGCCGCCCTTCGCGCCCTGGGCCATCTCCCTCCCAGCCGGTCCCCGCCGCCGCCGCCCTTCGCGCCCTGGGCCATCTCCCTCCCACCTCCCTCCGCGGAGCAGCCAGACAGCGAGGGCCCCGGCCGGGGGCAGGGGGGACGCCCCGTCCGGGGCACCCCCCCGGCTCTGAGCCGCCCGCGGGGCCGGCCTCGGCCCGGAGCGGAGGAAGGAGTCGCCGAGGAGCAGCCTGAGGCCCCAGAGTCTGAGACGAGCCGCCGCCGCCCCCGCCACTGCGGGGAGGAGGGGGAGGAGGAGCGGGAGGAGGGACGAGCTGGTCGGGAGAAGAGGAAAAAAACTTTTGAGACTTTTCCGTTGCCGCTGGGAGCCGGAGGCGCGGG(GTCTCAGGGGAATGGTAATAGTTTCTAATTCTTATATTT)GACCTCTTGGCGCGACGCTGCCCCGCGAGGAGGCAGGACTTGGGGACCCCAGACCGCCTCCCTTTGCCGCCGGGGACGCTTGCTCCCTCCCTGCCCCCTACACGGCGTCCCTCAGGCGCCCCCATTCCGGACCAGCCCTCGGGAGTCGCCGACCCGGCCTCCCGCAAAGACTTTTCCCCAGACCTCGGGCGCACCCCCTGCACGCCGCCTTCATCCCCGGCCTGTCTCCTGAGCCCCCGCGCATCCTAGACCCTTTCTCCTCCAGGAGACGGATCTCTCTCCGACCTGCCACAGATCCCCTATTCAAGACCACCCACCTTCTGGTACCAGATCGCGCCCATCTAGGTTATTTCCGTGGGATACTGAGACACCCCCGGTCCAAGCCTCCCCTCCACCACTGCGCCCTTCTCCCTGAGGACCTCAGCTTTCCCTCGAGGCCCTCCTACCTTTTGCCGGGAGACCCCCAGCCCCTGCAGGGGCGGGGCCTCCCCACCACACCAGCCCTG

3

AGTTTCCCTATCTGTAAATTGGGGACAGTAAATGTATGGGGTCGCAGGGTGTTGAGTGACAGGAGGCTGCTTAGCCACATGGGAGGTGCTCAGTAAAGGAGAGCAATTCTTACAGGTGTCTGCCTCCTGACCCTTCCATCCTTCAGGTGTCCTGTTGCCCCCTCCTCCCACTGACACCCTCCGGAGGCCCCCATGTTGACAGACCCTCTTCTCCTACCTTGTTTCCCAGCCTGACTCTCCTTCCGTTCTGGGTCCCCCTCCTCTGGTCGGCTCCCCTGTGTCTCATCCCCCGGATTAAGCCTTCTCCGCCTGGTCCTCTTTCTCTGGTGACCCACACCGCCCGCAAAGCCACAGCGCATCTGGATCACCCGCTTTGGTGGCGCTTGGCCGCCAGGAGGCAGCACCCTGTTTGCGGGGCGGAGCCGGGGTGCCCGCCCCCTTTCCCCCAGGGCTGAAGGGACCCCCCTCGGAGCCCGCCCACGCGAGATGAGGACGGTGGCCCAGCCCCCCCATGCCCTCCCCCTGGGGGCCGCCCCCGCTCCCGCCCCGTGCGCTTCCTGGGTGGGGCCGGGGGCGGCTTCAAAACCCCCTGCCGACCCAGCCGGTCCCCGCCGCCGCCGCCCTTCGCGCCCTGGGCCATCTCCCTCCCAGCCGGTCCCCGCCGCCGCCGCCCTTCGCGCCCTGGGCCATCTCCCTCCCACCTCCCTCCGCGGAGCAGCCAGACAGCGAGGGCCCCGGCCGGGGGCAGGGGGGACGCCCCGTCCGGGGCACCCCCCCGGCTCTGAGCCGCCCGCGGGGCCGGCCTCGGCCCGGAGCGGAGGAAGGAGTCGCCGAGGAGCAGCCTGAGGCCCCAGAGTCTGAGACGAGCCGCCGCCGCCCCCGCCACTGCGGGGAGGAGGGGGAGGAGGAGCGGGAGGAGGGACGAGCTGGTCGGGAGAAGAGGAAAAAAACTTTTGAGACTTTTCCGTTGCCGCTGGGAGCCGGAGGCGCGGGGACCTCTTGGCGCGACGCTGCCCCGCGAGGAGGCAGGACTTGGGGACCCCAGACCGCCTCCCTTTGCCGCCGGGGACGCTTGCTCCCTCCCTGCCCCCTACACGGCGTCCCTCAGGCGCCCCCATTCCGGACCAGCCCTCGGGAGTCGCCGACCCGGCCTCCCGCAAAGACTTTTCCC(GGGGAAA)CAGACCTCGGGCGCACCCCCTGCACGCCGCCTTCATCCCCGGCCTGTCTCCTGAGCCCCCGCGCATCCTAGACCCTTTCTCCTCCAGGAGACGGATCTCTCTCCGACCTGCCACAGATCCCCTATTCAAGACCACCCACCTTCTGGTACCAGATCGCGCCCATCTAGGTTATTTCCGTGGGATACTGAGACACCCCCGGTCCAAGCCTCCCCTCCACCACTGCGCCCTTCTCCCTGAGGACCTCAGCTTTCCCTCGAGGCCCTCCTACCTTTTGCCGGGAGACCCCCAGCCCCTGCAGGGGCGGGGCCTCCCCACCACACCAGCCCTG

4 AGTTTCCCTATCTGTAAATTGGGGACAGTAAATGTATGGGGTCGCAGGGTGTTGAGTGACAGGAGGCTGCTTAGCCACATGGGAGGTGCTCAGTAAAGGAGAGCAATTCTTACAGGTGTCTGCCTCCTGACCCTTCCATCCTTCAGGTGTCCTGTTGCCCCCTCCTCCCACTGACACCCTCCGGAGGCCCCCATGTTGACAGACCCTCTTCTCCTACCTTGTTTCCCAGCCTGACTCTCCTTCCGTTCTGGGTCCCCCTCCTCTGGTCGGCTCCCCTGTGTCTCATCCCCCGGATTAAGCCTTCTCCGCCTGGTCCTCTTTCTCTGGTGACCCACACCGCCCGCAAAGCCACAGCGCATCTGGATCACCCGCTTTGGTGGCGCTTGGCCGCCAGGAGGCAGCACCCTGTTTGCGGGGCGGAGCCGGGGTGCCCGCCCCCTTTCCCCCAGGGCTGAAGGGACCCCCCTCGGAGCCCGCCCACGCGAGATGAGGACGGTGGCCCAGCCCCCCCATGCCCTCCCCCTGGGGGCCGCCCCCGCTCCCGCCCCGTGCGCTTCCTGGGTGGGGCCGGGGGCGGCTTCAAAACCCCCTGCCGACCCAGCCGGTCCCCGCCGCCGCCGCCCTTCGCGCCCTGGGCCATCTCCCTCCCAGCCGGTCCCCGCCGCCGCCGCCCTTCGCGCCCTGGGCCATCTCCCTCCCACCTCCCTCCGCGGAGCAGCCAGACAGCGAGGGCCCCGGCCGGGGGCAGGGGGGACGCCCCGTCCGGGGCACCCCCCCGGCTCTGAGCCGCCCGCGGGGCCGGCCTCGGCCCGGAGCGGAGGAAGGAGTCGCCGAGGAGCAGCCTGAGGCCCCAGAGTCTGAGACGAGCCGCCGCCGCCCCCGCCACTGCGGGGAGGAGGGGGAGGAGGAGCGGGAGGAGGGACGAGCTGGTCGGGAGAAGAGGAAAAAAACTTTTGAGACTTTTCCGTTGCCGCTGGGAGCCGGAGGCGCGGGGACCTCTTGGCGCGACGCTGCCCCGCGAGGAGGCAGGACTTGGGGACCCCAGACCGCCTCCCTTTGCCGCCGGGGACGCTTGCTCCCTCCCTGCCCCCTACACGGCGTCCCTCAGGCGCCCCCATTCCGGACCAGCCCTCGGGAGTCGCCGACCCGGCCTCCCGCAAAGACTTTTCCCCAGACCTCGGGCGCACCCCCTGCACGCCGCCTTCATCCCCGGCCTGTCTCCTGAGCCCCCGCGCATCCTAGACCCTTTCTCCTCCAGGAGACGGATCTCTCTCCGACCTGCCACAGATCCCCTATTCAAGACCACCCACCTTCTGGTACCAGATCGCGCCCATCTAGGTTATTTCCGTGGGATACTGAGACACCCCCGGTCCAAGCCTCCCCTCCACCACTGCGCCCTTCTCCCTGAGGACCTCAGCTTTCC(GGGAATGTTTCGCAGTCTCACAAAAATTGAACCTAAGAAAAGAACAACAGTATAAAGGAGAAAGCTTCAAGACTA)CTCGAGGCCCTCCTACCTTTTGCCGGGAGACCCCCAGCCCCTGCAGGGGCGGGGCCTCCCCACCACACCAGCCCTG
