# Supplementary material for: Calcium-sensing stromal interaction molecule 2 upregulates nuclear factor of activated T cells 1 and transforming growth factor-β signaling to promote breast cancer metastasis
Source: Breast Cancer Res. 2019 Aug 29;21:99. doi: 10.1186/s13058-019-1185-1 (PMC6716836; doi:10.1186/s13058-019-1185-1)
Supplement: Supplementary file 2 — IACUC Aprroval Document for Mouse Breast Cancer Xenograft Tumor Assessments. (PDF 643 kb) [file 13058_2019_1185_MOESM2_ESM.pdf]

南方医科大学 Southern Medical University

实验动物伦理审查决议书 Decision of Laboratory animal ethics

决议编号/Resolution No.	L2015122	决议时间/Date of Resolution	2016.1.8
▲项目名称/Title of Project	Stim1 与 stim2 在乳腺癌转移过程中作用的研究		
申请时间/Date of Application			
▲课题组负责人/Principal Investigator	蔡春青		
是否通过初审/Trial Result	☐ √通过 /Pass ； ☐ 不通过 /Fail		
表决委员名单/The Committee Member List	顾为望、王元占、黎诚耀、彭鸿娟、周宏伟		
表决形式/Voting Form	☐ 会议表决/conference vote； ☐ √通讯表决/ correspondence vote		
表决情况/Voting	有效表决人数/The number of valid voting：__5__人；<br>建议批准实验人数 /The number of approval voting：__4__人；<br>建议调整方案后批准实验人数 /The voting number of approval after revision：__0__人；<br>建议不予批准人数/The number of disapproval voting：__1__人。		
审查决议/Decision of Censor	经伦理委员会审查：Under investigation of Ethic Committee:<br>☐ √同意/Approval； ☐ 不同意/Disapproval；<br>☐ 修改后同意实施实验方案/Approval after revision。		
IACUC 批准号/ IACUC Approval No.			
IACUC 主任或授权人签名 /Signature of IACUC Chief or Authorized Person	[signature]<br>时间/Date: 2016年1月9日	南方医科大学实验动物伦理委员会(章)/ Southern Medical University Experimental Animal Ethics Committee(stamp)	

注：标注“▲”由申请人填写。
