# Supplementary material for: Calcium-sensing stromal interaction molecule 2 upregulates nuclear factor of activated T cells 1 and transforming growth factor-β signaling to promote breast cancer metastasis
Source: Breast Cancer Res. 2019 Aug 29;21:99. doi: 10.1186/s13058-019-1185-1 (PMC6716836; doi:10.1186/s13058-019-1185-1)
Supplement: Supplementary file 3 — IRB Approval Document for Human Breast Cancer Tissue Microarray Assessments. (PDF 2603 kb) [file 13058_2019_1185_MOESM3_ESM.pdf]

河南省通许县人民医院伦理委员会审核意见书

项目名称	人体组织生物样本采集		
任务来源	陕西超英生物科技有限公司（完成国家863计划项目）	项目编号	
承办单位	通许县人民医院	项目负责人	李军、步雪

审核结果

参加伦理委员会会议 6 人，投票结果：

1，同意 6 票；　　2，做必要的修正后同意____票；

3，不同意____票；　　4，做必要的修正后再议____票；

5，终止____票；　　6，暂停已批准的实验____票；

伦理委员会审查意见：

1，　该项目的知情同意书符合项目规范，知情同意程序合理。

2，　此项目的实施不增加患者的临床风险。

3，　此项目收集的标本，仅被用于科研项目，或经相关主管部门审核通过的国际国内相关产品的研发合作项目，而非不合规范的商业用途。

4，　病人的隐私权在项目设计中得到了充分的保护。

记录人签名：[illegible]　　主任委员会签名：[illegible]

通许县人民医院 医学伦理委员会

2004年5月10日　　2004年5月10日
